# Selective occlusion of the hepatic artery and portal vein improves liver hypertrophy for staged hepatectomy

**DOI:** 10.1186/s12957-019-1710-9

**Published:** 2019-10-07

**Authors:** Changku Jia, Ke Ge, Sunbing Xu, Ling Liu, Jie Weng, Youke Chen

**Affiliations:** 1 Department of Hepatobiliary Pancreatic Surgery, Affiliated Hangzhou First People’s Hospital, Zhejiang University School of Medicine, Zhejiang Clinical Research Center of Hepatobiliary and Pancreatic Diseases, No. 261, Huansha Road, Hangzhou, 310006 China; 2 Department of Hepatobiliary Pancreatic Surgery, The First Affiliated Hospital of Hainan Medical College, Haikou, 570102 China

**Keywords:** Staged hepatectomy, Portal vein ligation, Hepatic artery ligation, Future liver remnant, Hepatocellular carcinoma

## Abstract

**Background:**

To evaluate the safety and feasibility of selective occlusion of the hepatic artery and portal vein (SOAP) for staged hepatectomy (SOAPS) in patients with hepatocellular carcinoma (HCC)

**Methods:**

From December 2014 to August 2018, 9 patients with unresectable HCC were chosen to undergo SOAPS. SOAP without liver partition was performed in the first stage. The second stage was performed when future liver remnant (FLR) was equal to or bigger than 40% of the standard liver volume (SLV). The growth rate of FLR, perioperative outcomes, and survival data was recorded.

**Results:**

In the first stage, all the 9 patients completed SOAP. Two cases received radiological interventional method and 7 cases received open operation. None of them developed liver failure and died following SOAP. After SOAP, FLR increased 145.0 ml (115.0 to 210 ml) and 37.1% (25.6 to 51.7%) on average. The average time interval between the two stages was 14.1 days (8 to 18 days). In the second stage, no in-hospital deaths occurred after SOAPS. One patient suffered from liver failure after SOAPS, and artificial liver support was adopted and his total bilirubin level returned to normal after postoperative day 35. The alpha-fetoprotein level of 8 patients reduced to normal within 2 months after SOAPS. Among 9 patients, 5 patients survived, 4 patients died of intrahepatic recurrence, lung metastasis, or bone metastasis. In the 5 survived cases, bone metastasis and intrahepatic recurrence were found in 1 patient, intrahepatic recurrence was found in another patient, and the remaining 3 patients were free of recurrence. The median disease-free survival time and overall survival time were 10.4 and 13.9 months, respectively.

**Conclusion:**

SOAP can facilitate rapid and sustained FLR hypertrophy, and SOAPS is safe and effective in patients with unresectable HCC.

## Highlights

1. SOAP can facilitate rapid and sustained FLR hypertrophy.
2. SOAPS is safe and effective in patients with unresectable HCC.

## Background

Liver resection, a curative therapy, is the most successful treatment for hepatocellular carcinoma (HCC) in appropriate stages [1]. The Barcelona Clinic Liver Cancer (BCLC) staging system was worldwide accepted. According to the BCLC system, the HCCs larger than 10 cm and/or with portal vein tumor thrombus are not suggested to surgical treatment [2]. But the shortcoming of the BCLC system might keep many patients off benefiting from liver resection when the lesions were staged as intermediate (B) and advanced (C). A lot of studies [3–5] reported favorable outcomes of liver resection compared with the nonsurgical options. Based on the previous evidences, some radical opinions were proposed that liver resection could be offered to any HCCs > 5 cm, as long as negative margin and appropriate liver function could be achieved [6]. Commonly, the liver function might be the key limiting factor for liver resection, particularly the postoperative liver function. Liver function is based on adequate liver volume. For HCCs, residual volume of 20% was acceptable in normal liver, but as much as 30% in chronic liver disease without cirrhosis and 40% in Child A cirrhosis were required for a safe liver resection [7]. Insufficient future liver remnant (FLR) would result in liver failure (LF) which is a fatal complication. When facing insufficient FLR, transcatheter arterial chemoembolization (TACE) is an alternative [8]. However, the incidence of local tumor recurrence after TACE is higher than that reported after surgical resection [8, 9]. Moreover, the long-term survival after TACE was much less than that after hepatectomy [5]. Thus, resection of large HCCs, which were defined as inoperable previously, became another option. In 1990, Makuuchi et al. [10] reported FLR hypertrophy achieved by portal vein embolization (PVE), which was known as the first-stage operation of conventional staged hepatectomy (CSH). The CSH extended the surgical indication for unresectable hepatic cancer. However, the major disadvantage of CSH was the long time interval between stages. Following portal vein ligation (PVL) or PVE, a 40% volume increase of FLR took at least 3 to 8 weeks [11]. Moreover, approximately 30% of patients who underwent CSH could not complete the second stage due to low hypertrophy efficiency [11].

In 2012, Schnitzbauer et al. [12] described a novel approach—associating liver partition and portal vein ligation for staged hepatectomy (ALPPS), which resulted in a hepatic volume increase of 47–93% in 6–14 days [13]. The shorter time interval of ALPPS caused a higher completion rate for staged hepatectomy, as high as 95–100% [13]. However, it was unacceptable that ALPPS unfortunately led to a morbidity rate of 68% and a mortality rate of 14% [12].

The underlying mechanisms behind the rapid growth of FLR were postulated that the effect relied on the discontinuation of portal circulation after PVE and transection between the normally perfused and deportalized liver parts [14, 15]. Based on these studies, we hypothesized that the FLR hypertrophy would be promoted after redistributions of arterial circulation and portal circulation. In the present study, we introduce a novel and safe method of selective occlusion of the hepatic artery (HA) and portal vein (PV) for staged hepatectomy (SOAPS) which balances surgical safety and growth effectiveness and consists of two stages. In the first stage, selective occlusion of the hepatic artery and portal vein (SOAP) without liver partition is performed. There are two approaches that could be selected to conduct SOAP, which are interventional method and open surgery. The former is preferred along with patient’s consent and technical feasibility. In the second stage, right trisectionectomy, right hemihepatectomy, or left trisectionectomy are performed respectively if the FLR has increased sufficiently.

## Methods

### Patients

Between December 2014 and August 2018, 9 consecutive patients in our center underwent this novel procedure, including 6 males and 3 females. Their average age was 43.9 years. All patients carried overexpressed alpha-fetoprotein (AFP) in serum and were diagnosed with HCC. The average tumor diameter was 128.4 mm. Five patients had satellite lesions and two patients had a cancer embolus in the right PV branch. The average FLR volume (FLRV) was 400.4 mL, and the ratio of FLRV to the standard liver volume (SLV) was 32.7% on average before the first stage. The SLV was estimated using the method reported by Urata et al. [16]. As all patients are suffering from chronic hepatitis B, at least 40% of SLV was considered as sufficient for the FLR. Patients’ characteristics are summarized in Table 1. According to tumor size and location, 6 patients were scheduled to right hemihepatectomy, 2 patients were right trisectionectomy, and 1 patient was left trisectionectomy. The planned surgeries were listed in Table 1. The terminology of liver resections was according to the Brisbane 2000 Nomenclature of Liver Anatomy and Resections [17].

**Table 1:** Characteristics of patients and tumor

Variable	Age (years)	Gender	Tumor diameter (mm)	AFP (ng/ml)	Child’s score	Planned procedure	Initial FLRV (ml), % of the SLV
Patient 1	23	Female	114	> 2000	5	Right trisectionectomy	421, 32.2%
Patient 2	42	Female	89	> 2000	5	Right hemihepatectomy	312, 28.7%
Patient 3	46	Male	123	582.7	5	Right hemihepatectomy	350, 25.0%
Patient 4	54	Male	155	557.9	6	Right hemihepatectomy	511, 37.9%
Patient 5	51	Male	130	1994.4	6	Left trisectionectomy	505, 35.8%
Patient 6	52	Female	95	227.5	6	Laparoscopic right hemihepatectomy	456, 37.7%
Patient 7	41	Male	205	> 2000	6	Right trisectionectomy	306, 27.3%
Patient 8	44	Male	130	1290.4	5	Right hemihepatectomy	385, 35.4%
Patient 9	42	Male	115	850.8	6	Laparoscopic right hemihepatectomy	358, 34.5%

*AFP* the level of serum alpha-fetoprotein before operation, *FLRV* the volume of the future liver remnant, *Initial FLRV*, the FLRV before the first-stage operation, *SLV* standard liver volume

This study was approved by the ethics committee of our centers (2014KYNo.017). All patients were told the purpose of this study and signed the informed consents. The Child-Pugh Score was adopted for preoperative liver function evaluation and combining Child-Pugh Score and FLRV for predicting postoperative liver dysfunction [18]. The comprehensive complication index (CCI) was adopted for evaluating postoperative morbidity [19]. Postoperative LF was assessed by 50-50 criteria [20]. The FLRV was evaluated by contrast-enhanced computed tomography (CT) nearly before SOAP and a week after SOAP, if the FLRV did not grow to an enough volume, the additional CT scan would be done a week later. Volumetric data were obtained from the portal phase image. We calculated FLRV from CT film by the method reported by Yoo et al. [21]. The gallbladder and hepatic veins were defined as the borderlines among different liver lobes. The time interval between the two stages was defined as below: The terminal point of “time interval” was the time of the satisfactory CT scan (the “second” CT scan), which contained a sufficient FLRV after SOAP. Then, the planned hepatectomy would be done nearly after the time of this CT scan. The starting point of “time interval” was the time of SOAP. The “first” CT scan would be done nearly before the time of SOAP, commonly less than a week. The growth rate of FLRV = (FLRV of the “second” CT scan–FLRV of the “first” CT scan)/time interval. The FLRV change was expressed as growth range, which was calculated via the formula: growth range = (FLRV after operation–FLRV before operation)/FLRV before operation × 100%.

### Surgical and interventional procedure

In the current study, SOAPS consisted of two stages. SOAP without liver partition was performed in the first stage. Hepatectomy was then performed in the second stage if the FLRV increased sufficiently. The two-stage operations were sequentially performed in one hospital stay.

In the first stage, 7 patients underwent a surgical procedure and 2 patients underwent an interventional procedure. The latter procedure was preferred in this study. But the performance of the interventional procedure was based on the patient’s consent and the feasibility assessment. The assessment was from the discussion between surgeons and radiologists according to the preoperative CT film and ultrasonography. If the path of PV puncture was too closed to or covered by the tumor, the percutaneous PVE became impossible. The 9 patients were treated by the same chief surgeon, but in the two different centers. The patients 1–5 received treatment at the First Affiliated Hospital of Hainan Medical College. The patients 6–9 were treated at the Affiliated Hangzhou First People’s Hospital, Zhejiang University School of Medicine. The patient 6 and patient 9 were the only two patients who underwent interventional first-stage procedure.

**Fig. 1:**
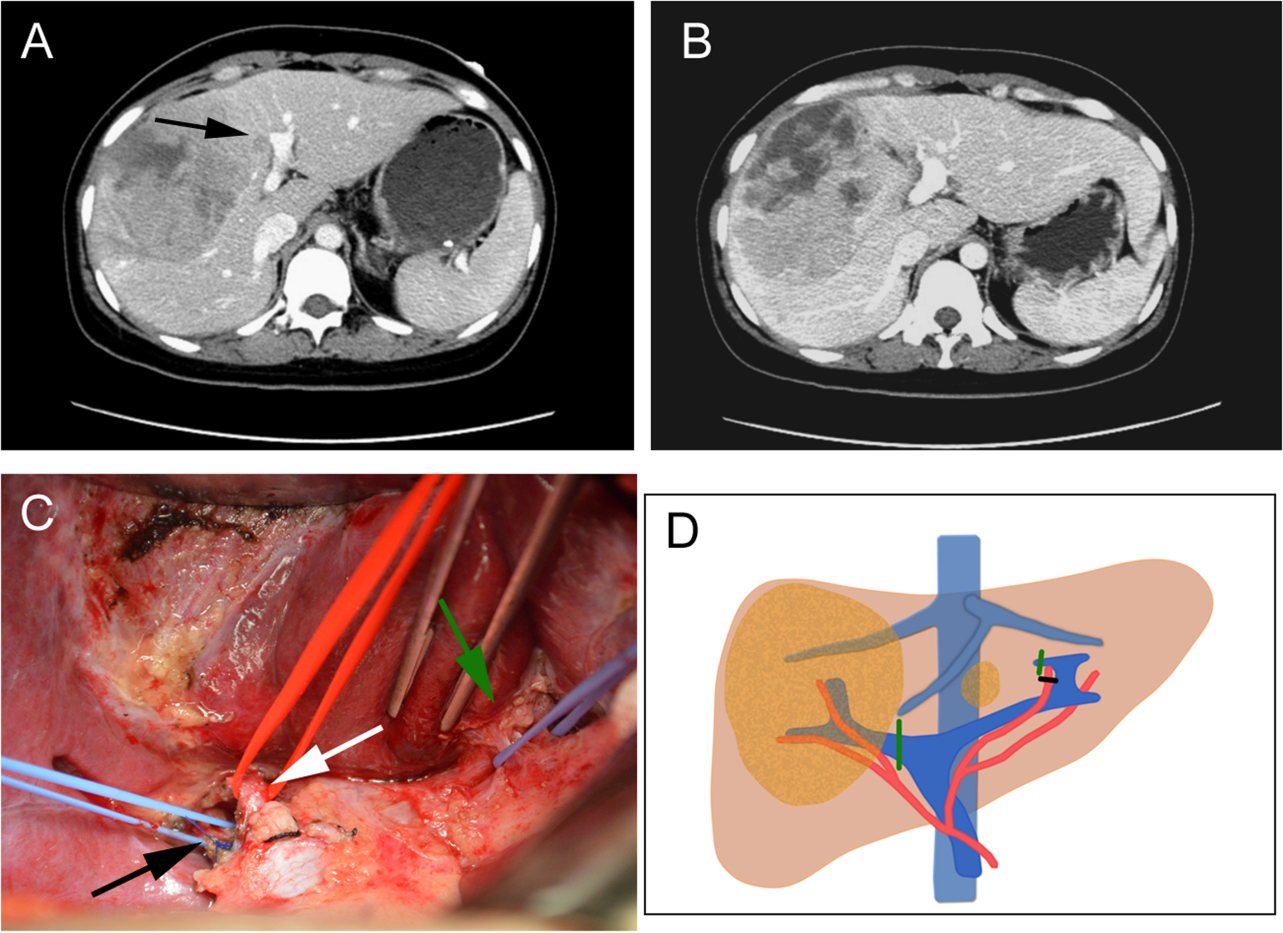
Preoperative findings and operative schema of patient 1 during the first stage. **a** Preoperative contrast CT revealed a huge hepatocellular carcinoma with a satellite lesion (arrow) in the right and left medial lobe of liver. **b** The contrast CT showed an obvious volume increase of left lateral lobe on the postoperative day 12. **c** After the extraparenchymal separation, the main branch of right PV (black arrow), the main branch of right HA (white arrow), and the vascular bundle (including PV and HA) feeding to left medial lobe (green arrow) were exposed. **d** Operative schema of the first stage. Both main branch of right PV and PV feeding to left medial lobe were ligated (green line); the main branch of HA feeding to segment 4 was ligated (black line).

**Fig. 2:**
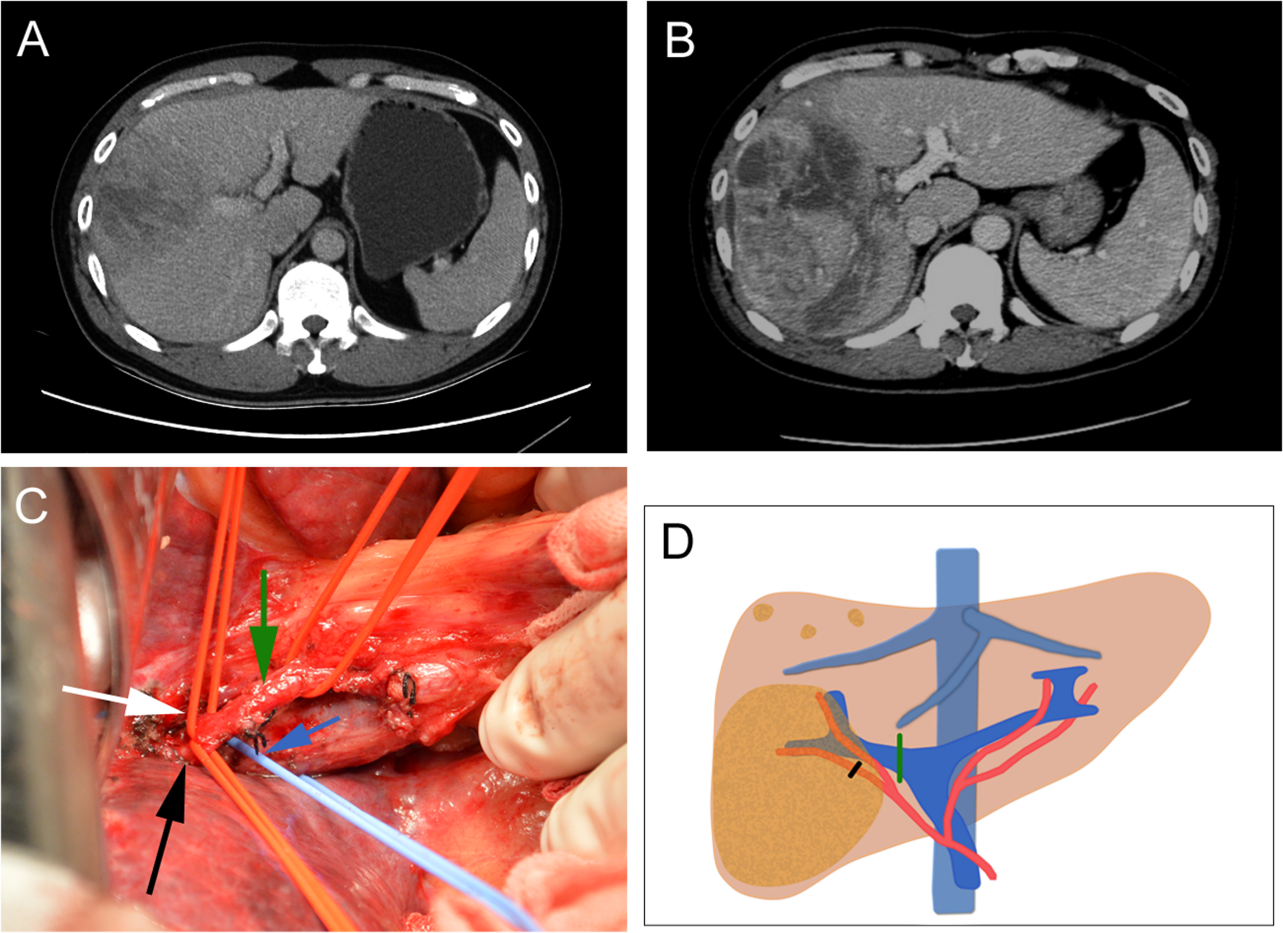
Preoperative findings and operative schema of patient 3 during the first stage. **a** Preoperative contrast CT revealed a huge hepatocellular carcinoma in the right lobe of liver. **b** The contrast CT showed an obvious volume increase of the future liver remnant on the postoperative day 13. **c** After the extraparenchymal separation, the main branch of right PV (blue arrow), the main branch of right HA (green arrow), the secondary arterial branches of the right anterior lobe (white arrow), and the right posterior lobe (black arrow) were exposed. **d** Operative schema of the first stage. The main branch of right PV was ligated (green line); the arterial branch of the right posterior lobe was ligated (black line)

For the patients who underwent surgical procedure, a laparotomy using a right subcostal incision was performed. The hepatic hilar region and Glisson’s capsule were separated to identify the main branches of the PV and HA. The main branches of the PV and HA were identified as ligation candidates. The right PV, left medial branch of PV, and HA were ligated in the patients scheduled to undergo right trisectionectomy in the second stage (Fig. 1). The right PV and right posterior or anterior branch of HA were ligated in the patients scheduled to undergo right hemihepatectomy in the second stage. Whether the right posterior or anterior branch of the HA was ligated was determined by the location of the tumor and the portion of normal liver tissue. The arterial branch mainly feeding the tumor-free lobe was ligated, and the arterial branch mainly feeding the tumor-bearing lobe was selectively reserved (Fig. 2). The left PV, right anterior branch of PV, and right anterior branch of HA were ligated in the patients scheduled to undergo left trisectionectomy in the second stage. All operations were performed without parenchymal transection. No hepatic ligaments were dissected and no drainage tube placement was carried out in this stage of the operation.

In the first stage, two patients were performed interventional therapies, including concomitant embolization of right PV and right anterior branch of HA. Candidate occluded vasculature is summarized in Table 2.

**Table 2:** Surgical or interventional procedures for first-stage operation, outcomes post first-stage operation

Variable	PV branches occluded	HA branches occluded	Procedure	Peak ALT (U/L)	Peak TBiL (μmol/L)	CCI
Patient 1	Right branch, left medial branch	Left medial branch	Right trisectionectomy	187	22.2	8.7
Patient 2	Right branch	Right posterior branch	Right hemihepatectomy	846.6	21.3	8.7
Patient 3	Right branch	Right posterior branch	Right hemihepatectomy	4620	39.6	8.7
Patient 4	Right branch	Right posterior branch	Right hemihepatectomy	676.9	35.5	27.6
Patient 5	Left branch, right anterior branch	Right anterior branch	Left trisectionectomy	1868.8	37.4	8.7
Patient 6	Right branch	Right anterior branch	Laparoscopic right hemihepatectomy	1034	29.9	8.7
Patient 7	Right branch, left medial branch	Left medial branch	Right trisectionectomy	1170	39	8.7
Patient 8	Right branch	Right posterior branch	Right hemihepatectomy	1778.8	38.7	8.7
Patient 9	Right branch	Right anterior branch	Laparoscopic right hemihepatectomy	1156.3	28.5	8.7

*PV* portal vein, *HA* hepatic artery, *ALT* serum alanine transaminase, *TBiL* serum total bilirubin, *CCI* the comprehensive complication index

The selective transarterial embolization (TAE) and PVE were performed as the methods described in the previous studies [21, 22]. The selective TAE was conducted under local anesthesia and fluoroscopic guidance. After the tip of the catheter was placed selectively in the right anterior branch of HA, iodinized oil was injected under fluoroscopic control, followed by embolization with gelatin-sponge particles. PVE was performed after TAE under general anesthesia in the same day. Under ultrasonographic guidance, right PVE was achieved by using coils and gelatin-sponge particles.

In the second stage, a bilateral subcostal incision via the original incision in the first stage was made during laparotomy in 8 patients. The planned hepatectomy was then performed using an anterior approach as reported in previous studies [23, 24]. Laparoscopic right hemihepatectomy using an anterior approach was performed in 2 patients during the second stage.

### Statistical analysis

All analyses were performed using SPSS v20.0. Disease-free survival time (DFS) and overall survival time (OS) were estimated using the Kaplan-Meier survival curves. Median DFS and median OS were calculated from the date of diagnosis for patients. Patients were followed up to death, or they were censored on December 1, 2018. The CCI was calculated by the online calculator (http://www.assessurgery.com).

## Results

In the first stage of SOAP, 7 patients underwent selective ligation and 2 patients underwent successful interventional embolization of HA and PV. For those 7 patients underwent surgical procedure, the average operation time was 104 min (80–130 min) and the average blood loss was 108 mL (50–150 mL). All patients suffered from mild fever below 39 °C after postoperative day (POD) 3–4 and were cured by noninvasive cooling techniques (CCIs: 8.7). Generally, the ice packs or luke-warm water baths would be employed when the temperature was under 38.5 °C; otherwise, cooled intravenous fluids might be considered. Patient 4 had mild pleural effusion in the right chest cavity (CCI: 27.6), who had mild oxygen desaturation ranging from 94 to 96% according to the fingertip pulse oximeter without oxygen inhalation. Although there was no difficulty breathing and chest pain, the oxygen saturation returned to above 98% after extracting about 350 ml fluid through a puncture tube. Serum alanine transaminase (ALT) and serum total bilirubin (TBiL) were both markedly elevated after the first-stage procedure, while gradually returned to normal after reaching peak values. ALT reached a peak value of 1639.9 U/L (187–4620 U/L) on average after POD 1–3, and TBiL reached a peak value of 31.2 μmol/L (21.3–39.6 μmol/L) on average after POD 1–7 (Table 2). None of the patients developed LF and no deaths occurred. FLRV increased by 145.0 mL on average at a rate of 14.7 mL/day. The average ratio of FLRV to SLV increased from 32.7 to 45.1% after SOAP. The average time interval between the two stages was 14.1 days (8–18 days). The growth data was listed in Table 3.

**Table 3:** The growth data of the hepatic remnant after SOAP and the short-term and long-term outcomes after the second-stage operation

Variable	Terminal FLRV (ml), % of the SLV	Growth rate (ml/day)	Time interval (days)	Growth range (%)	CCI	AFP* (ng/ml)	LOS* (days)	DFS (months)	OS (months), status
Patient 1	550, 53.5%	18.4	12	30.60	8.7	15.9	19	4	9.2, dead
Patient 2	456, 41.9%	20.6	8	46.20	8.7	18.1	9	> 40.2	40.2, alive without disease
Patient 3	531, 37.7%	16.5	13	51.70	43.3	4.7	12	7	13.8, dead
Patient 4	642, 49.0%	10.9	15	25.60	8.7	8.5	26	3.6	9.5, dead
Patient 5	715, 54.1%	25.8	17	41.60	8.7	9.9	13	6.2	20.8, alive with disease
Patient 6	581, 43.8%	10.4	15	27.40	8.7	3.2	12	> 13.3	13.3, alive without disease
Patient 7	420, 37.8%	9	18	37.70	27.6	289.2	22	2.2	14.5, dead
Patient 8	516, 45.6%	10.9	13	34.00	8.7	14.8	14	6.5	13.5, alive with disease
Patient 9	498, 42.7%	10	16	39.10	8.7	19.3	18	> 10.5	10.5, alive without disease

*FLRV* the volume of the future liver remnant, *terminal FLRV* the FLRV before the second-stage operation, *growth rate* the kinetic growth rate of the FLRV, *time interval* the time interval between the two stages, *SLV* standard liver volume, *growth range* = (FLRV after operation—FLRV before operation)/FLRV before operation × 100%, *DFS* disease-free survival time, *OS* overall survival time, *CCI* the comprehensive complication index, AFP* the level of serum alpha-fetoprotein 2 months after the second-stage operation, LOS* the length of hospital stay after the second operation. The end of follow-up is December 1, 2018

In the second stage, all patients completed the planned hepatectomy. The average operation time was 260 min (180–420 min), and the average blood loss was 640 mL (200–1300 mL). All postoperative pathological diagnoses were HCC. Of these 9 cases, 3 cases were histologically identified as highly differentiated, 4 cases were moderately differentiated and 2 cases were poorly differentiated. There were 3 patients’ liver capsules involved by tumors. All the surgical margins were negative (> 1 mm). Vessel cancer emboli were found in 7 cases. No in-hospital deaths occurred after SOAPS. Patient 4 developed LF after SOAPS and artificial liver support was adopted for him and his TBiL was returned normal after POD 35 (CCI: 43.3). Intra-abdominal collection was found at POD 5 with fever in patient 7, which was drained by abdominal catheterization guided by ultrasound and was diagnosed with bile leakage (CCI: 27.6). All patients suffered from mild fever below 39 °C after POD 3–5 and were cured by noninvasive cooling techniques described above (CCIs: 8.7). The mean length of hospital stay after SOAPS was 16.1 days (9–26 days). The AFP level in 8 patients reduced to normal within 2 months after SOAPS. Of these 9 patients, 4 patients died of intrahepatic recurrence, lung metastasis, or bone metastasis. Five of them survived when censored on December 1, 2018, and the longest survival time was 40.2 months. Among the 5 survived cases, bone metastasis and intrahepatic recurrence were found in 1 patient 6.2 months after SOAPS; intrahepatic recurrence was found in another patient 6.5 months after SOAPS, and the remaining 3 patients were without recurrence. The DFS was 10.4 months, and the OS was 13.9 months. In this study, Sorafenib—the molecular targeted anti-tumor drug used for HCC, was given to the patients with metastasis or recurrence. The short-term and long-term outcomes were listed in Table 3.

## Discussion

For large HCCs, a sufficient resection margin is an independent protective factor for prognosis [25]. In addition, anatomic liver resection, rather than non-anatomic liver resection, is essential for long-term survival [26, 27]. Of note, complete anatomic hepatectomy for a large HCC commonly requires extensive liver resection. However, the inadequacy of FLRV has become an intractable clinical problem for hepatectomy. The incidence of LF after major hepatectomy ranged from 1.2 to 32.0%, which was related to 80% of postoperative mortality [28, 29]. Small FLRV was an independent predictor for postoperative LF [29]. Another important parameter was FLR function, which was limited by the underlying liver diseases such as cirrhosis and steatosis [30]. Thus, the requirement of FLRV could be relaxed in a healthy liver but must high as 40% of total liver volume (TLV) in cirrhosis [7, 29]. In our study, SLV was adopted to estimate the actual TLV, which was due to the followed reasons. First, the TLV had to be calculated from CT film after removing the tumor volume. However, the shape of tumor was not always regular, which would influence the accuracy of calculation. The error would be further enlarged with the appearance of satellite lesions. Second, SLV was estimated from body surface area, which was not influenced by the underlying liver disease and much closer to a healthy liver [31]. Adoption of FLRV/SLV ratio but not FLRV/TLV ratio actually raised the surgical criteria, because the TLV would be reduced due to liver disease such as cirrhosis [32, 33].

PVE, known as the first-stage operation of CSH, has a long time interval between treatment stages and that may elicit tumor progression. Moreover, approximately 30% of patients who underwent PVE could not accomplish the second stage of the operation owing to low hypertrophy efficiency [11]. For ALPPS, although it induces rapid hypertrophy of the FLR and cuts down the time interval between treatment stages, it unexpectedly results in high morbidity and mortality [12, 13, 34–36]. Hence, several modified procedures of the first stage were proposed to overcome the disadvantages of CSH and ALPPS. The liver splitting of ALPPS was the main concern about higher postoperative morbidity [37]. In case of total parenchymal transection, there were several variations of liver splitting, such as partial splitting and in situ splitting by tourniquet compression, radiofrequency ablation, or microwave ablation [38]. The former was so-called partial-ALPPS combining partial parenchymal transection and PVE in stage 1. Petrowsky et al. [39] reported that the partial liver splitting between 50% and 80% of total liver transection surface could achieve a comparable hypertrophy with APPPS (median 60% vs. 61%), and an absolutely lower severe complication rate (0% vs. 33%), but only one out of 24 patients was diagnosed of HCC. For HCC, Chan et al. [40] reported that partial-ALPPS could not gain as faster hypertrophy as ALPPS (17.5 vs. 31.2 mL/day). The tourniquet-ALPPS replaced the liver splitting with a tourniquet bound 1-cm deep in the surface around the liver transection line. However, the 64% morbidity and 9% mortality made this improvement unsatisfactory [41]. The first-stage procedures with radiofrequency ablation or microwave ablation were somewhat similar, which gained rapid hypertrophy but lower morbidity compared with ALPPS [42]. A recent randomized controlled trial (REBIRTH trial) of PVE versus ALPPS assisted with radiofrequency (RALPPS) reported that RALPPS could trigger a much faster hypertrophy and comparable morbidity compared with PVE [43]. Even ALPPS was reported to have a comparable surgical safety as PVE by a recent randomized controlled trial (Ligro trial) [44], which was completely opposite to the conclusions of many recent meta-analyses [45, 46]. However, most of the samples of these two randomized controlled studies were colorectal liver metastases. Thus, whether the conclusions remained stable for HCC was unclear. Guiu et al. [47] developed a novel procedure with simultaneous ipsilateral hepatic vein embolization and PVE, so-called liver venous deprivation technique, which resulted in a mean degree of hypertrophy of 12.7 % after mean 23 days. Out of 7 patients, the only patient with HCC gained a growth rate of 12.2 mL/day in this study. However, sequential portal and hepatic vein embolization revealed liver hypertrophy was very slow in some patients with cirrhosis or HCC [48, 49].

Herein, we introduce SOAPS—a novel and safe method with two-stage hepatectomy, which balances surgical safety and growth effectiveness. The HA and PV were selectively ligated or embolized without parenchymal transection and hepatic ligament dissection in the first stage. A drainage tube was even not needed in the first stage. Therefore, complications were significantly decreased compared with ALPPS, such as abdominal bleeding, adhesions, and bile leakage [38]. And even the oncological safety would be improved as the classical approach of ALPPS was criticized for its “all-touch” defect [50]. Additionally, no postoperative mortality occurred both in the first and second stage. SOAP induced satisfactory hypertrophy of the FLR. Previous evidences [46, 51] showed that the kinetic growth rate was 14.4–32.7 mL/day for ALPPS and 2.42–4.4 mL/day for CSH, and the time to reach a sufficient FLRV was 6–18 days for ALPPS and 20–168.8 days for CSH. In our study, FLR increased in all patients after SOAP and the FLRV increased by 145.0 mL on average. The average growth rate was 14.7 mL/day. The growth rate after SOAP was comparable to ALPPS and was much faster than CSH. Most importantly, considerable hypertrophy was achieved without liver partition.

Although with a shorter follow-up period, SOAPS had achieved a comparable survival result with ALPPS. D’Haese et al. [52] reported that the median OS was 5.9 months and the median DFS was 5.1 months in 35 patients with intermediate-stage HCC after ALPPS. In our study, the shortest DFS was 2.2 months and the longest DFS was observed in patient 2, who had no sign of recurrence during the follow-up period (40.2 months). The median DFS was 10.4 months, and 3 patients were alive without disease. Besides, the median OS was 13.9 months in our study. A recent study [5] from a single center in China reported that the HCC patients (tumor diameter range from 6 to 31 cm) after ALPPS gained the 1-year OS as much as 64.2%, which was similar to our result (6 out of 9 patients). But their study [5] complied with a 91.1% uncompleted rate and a 11.1% 90-day mortality, which did not occur in our relatively small size study.

With regard to the mechanism of liver hypertrophy after the first stage of SOAP, we found that besides the reported PV blood redistribution, HA blood redistribution was one of the mechanisms of liver hypertrophy. Particularly, it was observed in patient 4 who had a cancer embolus in right PV which caused total obstruction of right PV and blood redistribution to left PV before SOAP. Even though, FLR of the patient still increased adequately after SOAP. As well as the results of previous studies, sequential PVE and TACE had been successfully performed for HCC and gained a significant liver hypertrophy [21, 22, 53, 54].

Hypoxia-enhanced invasiveness is a major concern in HA occlusion [55]. Massive necrosis of the tumor is still an alarming event after PVE plus HA ligation for large HCCs [56]. Thus, occlusion of HA was limited to one lobe (right posterior branch, right anterior branch, or left medial branch) in the SOAP. To prevent tumor hypoxia, necrosis, and tumor lysis syndrome, the arterial branch mainly feeding the tumor-free lobe was occluded and the arterial branch mainly feeding the tumor-bearing lobe was selectively reserved [57]. Therefore, the effectiveness of FLR hypertrophy and surgical safety was balanced.

Concomitant occlusion of candidate vasculature by a transcatheter endovascular technique is less invasive than that of a surgical procedure. However, interventional techniques are not always appropriate in all cases. In the current study, PV and HA branch in the left medial lobe were occluded in patients who underwent right trisectionectomy. The vessel branches of left medial lobe are too tiny and various to percutaneously and transhepatically catheterize and embolize. Hence, interventional or surgical techniques should be individualized in different patients. Another reason was that when the large tumor located closed to or covered the path of puncture, the PVE became unsafe and impossible. Removing the satellite lesions at the first stage was a good choice for most HCCs, but the removing was not suitable for any cases. In our cases series, the tumor size was large on average. As reported by several studies [58, 59], the risk of tumor rupture increased with the increase of tumor size, and the fatal complications including liver failure after rupture were as high as 12–42%. Most of the satellite lesions in our cases were closed to the main tumor or deep in the liver or near the important intrahepatic structures, resection, or ablation of which were not easy and oncologically safe.

As a case series study, relatively small sample size was a major limitation of this study. Fortunately, the results of the 9 patients revealed highly consistency. As the sample size accumulated, the reliability of the conclusion might be more stable. Second, the first-stage operations of our cases showed a degree of inconsistency. As mentioned above, the interventional technique was not suitable for any cases. Open surgery was given to 7 patients and interventional procedure was given to last 2 patients. Although they were the different routes to the same summit, the stability of results would reduce to a certain extent. In fact, the interventional approach would be the major method for SOAP, because of its minimal invasiveness. However, the surgical approach (open or laparoscopy) will not disappear, which will be optional when the interventional approach facing technical obstacles. Reducing the trauma of the operation is another focus. Thus, the minimally invasive methods such as laparoscopy and robot-assisted operation might be applied for the selected patients in the future.

## Conclusion

SOAP can facilitate rapid and sustained FLR hypertrophy. SOAPS is safe and effective in patients with unresectable HCC.

## Data Availability

Data will be made available by the authors on request.
